# Cancer and Clinical Biochemistry

**DOI:** 10.1038/sj.bjc.6690031

**Published:** 1998-09-24


*British Journal of Cancer* (1999) **79**(1), 185–187
© 1999 Cancer Research Campaign

### Book Reviews

### Cancer and Clinical Biochemistry

Peter Pannall and Dusan Kotasek, Clinical Biochemistry in Medicine Series, Series editors Gwyn McCreanor and William Marshall, London: ACB Venture Publications, 1997, pp 136, £21, ISBN 090242909 4

This book gives an overview of a frequently neglected area of cancer that at best has had patchy coverage in the past, namely the relationship between the biochemical changes that occur in cancer patients resulting from an increasing and progressive tumour burden, increased secretion of inappropriate substances and the effects of cytotoxic therapy on biochemical homeostasis.

The book is divided into six chapters: The Nature of Malignancy, The Biochemical Effects of Tumour Growth, Tumours and Tumour Markers – General Considerations, Tumours and Tumour Markers – Applications, Paraneoplastic Syndromes and The Treatment of Cancer.

The presentation in this book is of a high standard, with very clear figures and tables, and the information is given in a logical order. Many of the aspects discussed are illustrated with interesting case histories that reinforce the information in the text. At the end of each chapter are up-to-date references that look useful and allow the reader to expand their knowledge base in the particular area that interests them. Although the style of writing is didactic, the authors give balanced arguments for their views or reasons for the statements they make, and you often feel that they are speaking from personal experience. Their message is clear and precise. The book also has a good index and there are very few typographical errors.

The book starts with an introduction to cancer which, although superficial, presents an up-to-date view of the field, bringing in factors such as proto-oncogenes/suppressor genes, DNA repair and inherited cancers. Also, at the end of the chapter the authors discuss the possibility of molecular biology tools being used to investigate cancer in the future. This chapter will be valuable for anyone who is a bit unsure about the nature of cancer or who is a bit rusty on recent developments in the field and would like to be brought up to speed.

In considering the biochemical effects of the tumour on the host (Chapter 2), the authors discuss both the effects of metastasis on specific organs, for example liver or bone involvement, as well as more generalized damage caused by a growing and increasing primary tumour mass. The release of tumour products and the host tissue response to injury are also considered. The examples and illustrations for this part are excellent.

Chapter 2 leads naturally into the first of the two chapters on tumour markers. The first provides a realistic overview of a field which, in the past, has generated numerous molecules that have claimed to be useful for monitoring cancer. Sensibly, the authors mainly restrict their comments to well-established markers such as CEA, alpha-fetoprotein, HCG and the so called ‘cancer antigens’ on mucins. Another focus of this chapter is a section on the interpretation of tumour markers, their limitations and the problems with assay variability. Standard topics such as sensitivity, specificity and receiver operating characteristic curves are also covered.

Chapter 4 is concerned with applications of specific tumour markers. This is approached by describing markers used to investigate tumours that arise in various body sites, for example gastrointestinal tract, breast, lung, ovary. This chapter is the longest in the book and is packed with information, giving ranges, specificity, sensitivity and recommendations for use on a wide range of cancer markers. Again, information is well organized and clearly presented with a number of interesting case histories.

The last two chapters of this book are directed more at the dedicated reader of the book rather than the casual browser. Chapter 5 is concerned with the paraneoplastic syndromes and would require a reasonable amount of previous knowledge in clinical biochemistry to obtain the maximum benefit from it. Many situations in which tumours produce inappropriate hormones are documented in detail. In chapter 6, the authors give an excellent summary of the cytotoxic chemotherapies that are available for cancer treatment and their mechanism of action. This leads to a final section that describes drug toxicity and how diagnostic biochemistry can help in monitoring these effects.

This book is not very long, 136 pages, but every page is packed with valuable information. Although it is aimed primarily at the professional clinical biochemist, its scope and clarity will appeal to a wide range of readers from the undergraduate student who is studying medicine or a medically related science through to the hospital oncologist.

*GA Turner,*
*Department of Clinical Biochemistry Medical School,*
*Framlington Place,*
*Newcastle-upon-Tyne NE2 4HH*

### Improving Care for Patients with Malignant Cerebral Glioma

Edited by E Davies and A Hopkins, pp 123

This is an excellent book which represents an outstanding summary of the current perspectives for the management of patients with glioma. It contains 12 chapters in 123 pages, and each of the chapters is appropriately referenced. An appendix is also included containing audit proformas for various issues of patient care for this condition. The book is a publication from a Royal College of Physicians Working Party on Malignant Cerebral Glioma and accompanies an article published by the same group in 1997 in *Br J Neurosurg* **11**, 318–330. In many ways, the book represents a more straightforward summary and contains additional information on the psychological aspects of care in this depressing condition.

There are important technical sections in the book. These include the issue of confirming the diagnosis and specific chapters dealing with surgical treatment, radiotherapy and chemotherapy. The issue of steroids is also discussed. There are resolved issues in the surgical treatment of glioma and regarding some of the aspects of radiotherapy. The issues are well described, and appropriate and helpful conclusions are drawn.

In the chapters dealing with the psychological aspects of the condition, there are chapters on breaking bad news, and one of these is written by the relative of a patient. This is written with considerable insight and has some extremely constructive points to make. From my personal point of view, I was disappointed that this chapter had relatively little to say about the period between surgical diagnosis and the commencement of radiotherapy treatment.

There are three chapters dealing with follow-up services and their organization, including one excellent chapter describing an alternative method of follow-up using a neuro-oncology nurse specialist and a 'telephone clinic'. It is noteworthy that this book supports the concept of follow-up at a time when this has been criticized from the perspective of a number of other tumour types in oncology.

The book also contains an interesting chapter on what general practitioners want to know about the condition, although, in my own experience, there is a considerable spectrum in the sort of information about the condition which individual GPs want or need to receive. There is a valuable chapter on palliative care, particularly in the community setting. The final chapter of the book is written from the perspective of a purchaser. This represents an interesting angle, and is very much more in line with the objectives of medical nursing and supportive care than one might otherwise have thought.

The final section of the book contains audit proformas for evaluating management, both of individual patients and treatment centres. There are several proformas looking at all aspects of patient care, including information giving, breaking bad news, technical aspects, follow-up details and management at time of relapse. These proformas have obviously been collated carefully. The reader is invited to photocopy these, because they are free from copyright, if they are considered helpful. Overall, I find this the weakest part of the book, purely because many of the proforma questions seem a little too superficial to reflect the very complex nature of this illness. Nevertheless, it is a valiant attempt to document what is or is not being achieved and can be used to assess the benefit of new strategies. It may well be that such information will help individual centres to develop their neuro-oncology service.

This book represents an outstanding summary of all aspects of the current clinical management of patients with malignant cerebral glioma. It is to be commended to anyone working in this field, including medical and surgical trainees, nurses, managers and purchasers. I hope the book will be revised from time to time, particularly in response to publication of important new information. Meanwhile, the book is extremely cheap considering its content, and should be on the bookshelf of all of us involved in the care of patients with malignant cerebral glioma.

*Neil Burnett*
*University of Cambridge*
*Box 193*
*Addenbrooke's Hospital*
*Cambridge CB2 2QQ*

### Apoptosis and Cancer

Edited by J Martin, Karger Landes Systems, pp 265

The process by which cancer cells may undergo a programmed cell death or apoptosis represents a 1990s burgeoning area of cancer research following on from the original seminal studies of Kerr and Wyllie in the early 1970s. This is reflected in the exponential increase in papers on apoptosis during the 1990s, numerous sessions at international cancer conferences and various books being devoted to the subject. This book, comprising 13 chapters from an international array of authors and 265 pages and published by Karger Landes Systems, is ambitious in attempting to compile, in a coherent manner, the latest developments. A little disappointing from a publishing viewpoint is the rather monochrome appearance of the book cover: a computer graphic of an apoptosing cancer cell or death pathway may have more immediate appeal to the casual bookshelf browser!

The early chapters ('Apoptosis and Cancer: an Overview' and 'Apoptosis: an Introduction' by SJ Martin, Maynooth, Ireland) serve as an appetizer to the later main courses and provide a sound introduction to the field and later, more specialized, contributions. Chapter 3, 'Apoptosis and Necrosis in Tumors', by N Toft and M Arends from the Wyllie Group in Edinburgh, attempts to clarify the roles of apoptosis versus necrosis in determining tumour growth (cell gain versus cell loss) and after chemotherapy. The role of angiogenesis and the potential of anti-angiogenesis therapy is mentioned. A minor quibble of Chapter 4 'Apoptosis and the Cell Cycle', by R Fotedar, L Diederich and A Fotedar (France), is the lack of a summarizing graphic of the cell cycle and where the various negative and positive regulators operate. Chapter 5, 'Bcl-2 Family Proteins: Role in Dysregulation of Apoptosis and Chemoresistance in Cancer', by J Reed (La Jolla), is comprehensive and excellent, and, for me, the highlight of the book. This chapter covers structural/sequence and, where known, functional details of the various positive and negative regulators of apoptosis within the bcl-2 family, their interactions, their role as determinants of chemoresponsiveness and chemoresistance and their prognostic significance in various cancer types. The chapter finishes with details of post-translational modifications of Bcl-2 (e.g. phosphorylation by paclitaxel) and possible strategies for inhibiting Bcl-2 function in cancers. Chapter 6 'Abl Tyrosine Kinase and the Control of Apoptosis' by G Amarante-Mendes and D Green (San Diego) describes the various members of the Abl family, how oncogenic forms such as Bcr-Abl confer resistance to apoptosis induction and the recent important observations connecting c-Abl to stress pathways (JNK/SAPK) after DNA damage. The role of p53 in apoptosis and cancer is covered in Chapter 7, by C Bellamy, R Malcomson and A Wyllie (Edinburgh). Although there are no illustrations, the chapter is comprehensive, including the role of p53 in apoptosis, growth arrest and DNA repair, p53 abnormalities in cancer and a little on the potential for p53 gene therapy. Following on from p53 is a consideration of the role of the retinoblastoma gene in the control of apoptosis (Chapter 8 by K Macleod and T Jacks, Cambridge, MA, USA). In particular, apoptosis via deregulation of the Rb-regulated transcription factor, E2F after loss of Rb is described.

Chapters 9–12 focus on apoptosis in a disease or organ setting: Programmed (apoptotic) Cell Death and Prostate Cancer (S Denmeade and J Isaacs, Johns Hopkins, Baltimore, USA) including apoptosis induced by androgen ablation therapy, a short chapter on Cell Death Regulation in the Kidney and in Renal

 © Cancer Research Campaign 1999

Neoplasms (T McDonnell, S Hewitt and G Saunders, MD Anderson, Houston, USA), Apoptosis in Leukemia (S McKenna and T Cotter, Cork, Ireland) and Cell Death in Neuroblastoma Tumors (G Melino et al, Rome, Italy). The final chapter (13) 'TNF-based Strategies for Manipulating Apoptosis: Adjuncts to Cancer Therapy' (G Wong, G Vehar and R Kaspar, San Francisco and UT, USA) provides an interesting insight into the possibility of protecting normal cells while sensitizing tumours using tumour necrosis factor (TNF) or lymphotoxin (LT) pretreatment.

Although many of the key players in this field have contributed chapters (e.g. A Wyllie, D Green, J Reed), it is perhaps surprising that there is no contribution from the Korsmeyer, Hickman/Dive or Evan groups. The strengths of the book lie in defining the key players and pathways, regulation and molecular pathology of apoptosis. A weakness is in the area of therapeutic exploitation of these findings. There is a general lack of coverage of new cancer drug development initiatives within the apoptosis arena (although admittedly much of this is closely guarded within the pharmaceutical industry and not yet within the public domain). However, for example, there is little mention of the ongoing bcl2 antisense trials in follicular lymphoma.

Overall, because of the rapidity of change in this cutting-edge field, some sections are inevitably already a little out of date; for example, discussions of the *ced4* human homologue Apaf-1 and the very recent $p16^{INK4}/p19^{ARF}$ products providing dual inactivation of the Rb and p53 pathways. Nevertheless, the book provides an excellent entry into the field for undergraduates, postgraduates or other cancer researchers for whom apoptosis in not their main area of interest. For the experts, the book is clearly no substitute for continuing to dip regularly into *Nature*, *Cell*, *Science*, etc.

*LR Kelland*
*Institute of Cancer Research*
*Clifton Avenue*
*Sutton*
*Surrey SM2 5PX*

© Cancer Research Campaign 1999

